# Smartphone Slit Lamp Imaging—Usability and Quality Assessment

**DOI:** 10.3390/diagnostics13030423

**Published:** 2023-01-24

**Authors:** Daniel Rudolf Muth, Frank Blaser, Nastasia Foa, Pauline Scherm, Wolfgang Johann Mayer, Daniel Barthelmes, Sandrine Anne Zweifel

**Affiliations:** 1 Department of Ophthalmology, University Hospital Zurich, University of Zurich, Frauenklinikstrasse 24, 8091 Zurich, Switzerland; 2 Department of Ophthalmology, University Hospital, LMU Munich, Mathildenstrasse 8, 80336 Munich, Germany

**Keywords:** imaging, slit lamp, photography, smartphone adapter, Custom Surgical, slit REC

## Abstract

Purpose: To assess the usability and image quality of a smartphone adapter for direct slit lamp imaging. Methods: A single-center, prospective, clinical study conducted in the Department of Ophthalmology at the University Hospital Zurich, Switzerland. The smartphone group consisted of 26 medical staff (consultants, residents, and students). The control group consisted of one ophthalmic photographer. Both groups took images of the anterior and the posterior eye segment of the same proband. The control group used professional photography equipment. The participant group used an Apple *iPhone 11* mounted on a slit lamp via a removable *SlitREC* smartphone adapter (Custom Surgical GmbH, Munich, Germany). The image quality was graded independently by two blinded ophthalmologists on a scale from 0 (low) to 10 (high quality). Images with a score ≥ 7.0/10 were considered as good as the reference images. The acquisition time was measured. A questionnaire on usability and experience in smartphone and slit lamp use was taken by all of the participants. Results: Each participant had three attempts at the same task. The overall smartphone quality was 7.2/10 for the anterior and 6.4/10 for the posterior segment. The subjectively perceived difficulty decreased significantly over the course of three attempts (Kendall’s W). Image quality increased as well but did not improve significantly from take 1 to take 3. However, the image quality of the posterior segment was significantly, positively correlated (Spearman’s Rho) with work experience. The mean acquisition time for anterior segment imaging was faster in the smartphone group compared to the control group (156 vs. 206 s). It was vice versa for the posterior segment (180 vs. 151 s). Conclusion: Slit lamp imaging with the presented smartphone adapter provides high-quality imaging of the anterior segment. Posterior segment imaging remains challenging in terms of image quality. The adapter constitutes a cost-effective, portable, easy-to-use solution for recording ophthalmic photos and videos. It can facilitate clinical documentation and communication among colleagues and with the patient especially outside normal consultation hours. Direct slit lamp imaging allows for time to be saved and increases the independence of ophthalmologists in terms of patient mobility and the availability of photographic staff.

## 1. Introduction

With the invention of the first usable ophthalmoscope in 1850 by Hermann von Helmholtz and with the development of the slit lamp in 1887 by Wilhelm von Zehender and Heinrich Westien and its improvements by Siegfried Czapski in 1897, Alvar Gullstrand in 1911, and Otto Henker in 1916, ophthalmology has experienced an uprising of examination possibilities of the anterior and posterior eye segment [1,2,3,4,5,6]. In terms of graphic documentation, Alfred Vogt was a pioneer of his time when he published in 1921 his atlas of meticulous drawings of findings he had observed via a slit lamp [7]. The accuracy of his drawings is impressive and most probably took a considerable amount of devotion and time, not to mention the skills necessary to produce such artworks. However, medical documentation by drawing is highly dependent on the skills and the subjective interpretation of the drawer. The increasing commercial availability of photography since its introduction in 1839 by Nicéphore Niépce and Louis-Jacques-Mandé Daguerre was quickly followed by the first attempts to objectively document slit lamp findings using photography [8]. The first attempts of slit lamp photography published in the literature date back to the late 1950s [9]. This technique was successively improved over the following years [10,11,12,13,14,15,16]. Attempts with Polaroid instant photography systems have been made, avoiding the time gap between image acquisition and photo development and fixation on paper [17,18]. Slit lamp photography was significantly facilitated when digital cameras became available and could be mounted to slit lamps in the early 2000s [19,20,21]. Integrated cameras by slit lamp manufacturers such as the Haag–Streit *Imaging Module 910* (Haag-Streit AG, Köniz, Switzerland) were released but carried an expensive price tag [6]. Since the invention of the “smartphone” whose distribution has experienced a boost with the introduction of the Apple *iPhone* (Apple Inc., Cupertino, CA, USA) series, nowadays, a digital camera with a telephone function is carried around in most pockets [22,23]. Simultaneous operation of the slit lamp and a smartphone camera that is held against the slit lamp eyepiece can be uncomfortable, especially during fundoscopy, with having to hold a diagnostic lens [24]. Therefore, a variety of adapters have been developed to mount smartphones to slit lamps [24,25,26,27]. A disadvantage that is in common with some adapters on the market is either specificity for one phone model or for one slit lamp type. Moreover, with the adapter attached to the slit lamp, normal, binocular operation of the slit lamp is not possible. Therefore, in our study, we introduced and evaluated a novel, universal smartphone slit lamp adapter that can be mounted to any eyepiece and can be easily flipped aside when binocular use of the slit lamp is wanted.

## 2. Methods

### 2.1. Ethics

An ethics waiver was issued by the local Ethics Committee of the Canton of Zurich (project number: BASEC-Nr. Req-2022-00763). The study adheres to the tenets of the 1964 Declaration of Helsinki and its later amendments.

### 2.2. Study Design

This is a single-center, prospective, clinical study conducted from July 2022 until September 2022 in the Department of Ophthalmology of the University Hospital of Zurich (USZ), Switzerland.

### 2.3. Participants

Two groups were established: a smartphone group and a control group. Included were only staff employed by the Department of Ophthalmology of the University Hospital of Zurich at that time.

### 2.4. Image Acquisition and Grading

**Figure 1:**
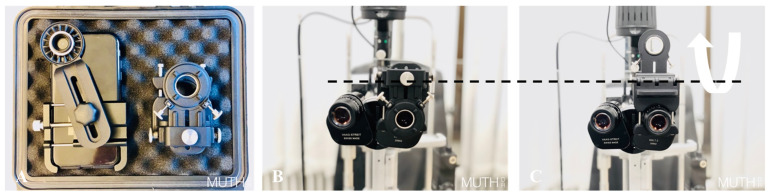
Custom Surgical *SlitREC* adapter: (**A**) smartphone adapter (left) and eyepiece adapter (right). (**B**) Eyepiece adapter mounted on a slit lamp via two hex socket screws (*Allen*/*Inbus* screws). The other two screws can be used optionally to hold the smartphone adapter. In our setting, the magnetic ring alone proved to be strong enough. (**C**) Eyepiece adapter flipped up to allow binocular use of the slit lamp.

**Figure 2:**
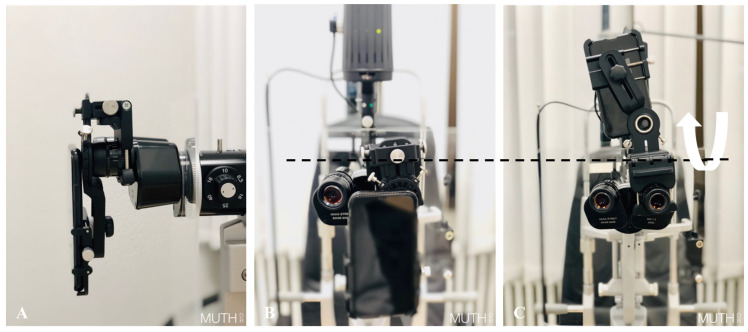
Smartphone mounted on a slit lamp via Custom Surgical *SlitREC* adapters: (**A**) adapter flipped down (capture mode), side view; (**B**) adapter flipped down (capture mode), front view; and (**C**) adapter flipped up (clinical mode) with a smartphone (held by magnets only), front view.

Both groups took images of the anterior segment and of the posterior segment of the same proband. The control group used their standard professional photo equipment (for the anterior segment: a Haag–Streit slit lamp camera *BX900* (Haag-Streit AG, Köniz, Switzerland), for the posterior segment: Zeiss *FF450+* (Carl Zeiss AG, Oberkochen, Germany). The smartphone group used an *iPhone 11*, (iOS v15.4, Apple Inc., Cupertino, CA, USA) mounted to a slit lamp (Haag-Streit *BQ900,* Haag-Streit AG, Köniz, Switzerland) via the removable *SlitREC* smartphone adapter set by Custom Surgical (Custom Surgical GmbH, Munich, Germany). The adapter set consists of two parts, a universal phone adapter that can be adjusted to any phone model and a universal eyepiece adapter that can be mounted to any slit lamp, laser device, or microscope (Figure 1A,B). Both adapters are connected via a magnet with optional screws for a more permanent setup (Figure 2A,B). The eyepiece adapter was designed to be left in place on the slit lamp. When it is not in use it can be flipped up or to the side and allows normal binocular usage of the slit lamp (Figure 1C and Figure 2C). For fundoscopy, the participants of the smartphone group all used the same Volk *78D* double aspheric non-contact lens (Volk Optical Inc., Mentor, OH, USA). Fundoscopy was carried out when pupil dilation was sufficient (≥4.0 mm diameter).

The dedicated camera application *MicroREC* (v.3.07 for Apple *iOS*) was used to take the photos. The acquisition time was defined as the mean time required to assemble the device, to take the picture, to disassemble the device, and to export the images via the application *imitoCam* (v3.8.2, imito AG, Zurich, Switzerland) to the content management software (CMS) *KISIM* (Cistec AG, Zurich, Switzerland). Each participant had three takes to perform the same task.

A usability questionnaire was taken by all of the participants asking for their subjective difficulty to perform the task at each take, ranging from 10/10 (very easy) to very difficult (0/10) (for an example see Supplementary Material SM 1). Furthermore, using the same questionnaire, work experience was judged by the years actively working in ophthalmology (Table 1). Smartphone experience was assessed by the time actively using a smartphone, the number of photos taken with a smartphone per year, and the subjective competence in taking private (non-ophthalmological) photos with a smartphone ranging from 10/10 (very confident) to 0/10 (not confident) (Table 1). The quality of all of the pictures was graded independently by two blinded ophthalmologists on a scale from 0/10 (low) to 10/10 (high quality) in comparison to the reference images by the professional photographer. The graders were blinded by the photographer. As we only had one reference image by one photographer, blinding to the device was not reasonably possible. The reference images were defined as having 10/10 quality. For the participants’ images, a quality threshold of ≥7/10 was considered by the authors as good as the reference images. Analogous as it is published in the literature about other imaging modalities, such as optical coherence tomography (OCT), a quality index (QI) of ≥7/10 should be sufficient to evaluate the relevant structures that allow an appropriate clinical interpretation. Participant images with a score ≥ 7/10 were considered as good as the reference images. Each image was graded in terms of image sharpness (focus), exposure, field of view (FOV), color, clinical interpretability, artifacts. The average of these subcategories generated an overall image quality score.

### 2.5. Statistical Analysis

Data were organized in Microsoft *Excel* (Microsoft Corp., Redmond, WA, USA) and statistically analyzed using *SPSS* software version 23 (v23, IBM Corp., Armonk, NY, USA), *R.app* (v4.1.0 GUI 1.76 for MacOS (The R Foundation for Statistical Computing c/o Institute for Statistics and Mathematics, Vienna, Austria), *RStudio* (RStudio PBC, Boston, MA, USA), and *StatPlus:mac* (v8.0.1.0 for MacOS, AnalystSoft, Walnut, CA, USA). Descriptive statistics such as the median and the interquartile range (IQR) were computed for the non-parametric data. We analyzed the data in regard to normal distribution using the Kolmogorow–Smirnow and the Shapiro–Wilk tests. We evaluated quality differences within the participant group between anterior segment and posterior segment images of the eye by calculating an asymptotic, two-tailed Mann–Whitey U (*z* value) test. To check for a possible learning curve with improvement of image quality, a Kendall’s *W* test was calculated to evaluate differences between take 1, take 2, and take 3. This was performed for the anterior segment and posterior segment photos. Furthermore, the mean time needed to take and export the image was calculated for the participant group and the control group. Within the smartphone group, the time differences between the three takes were evaluated by a Kendal’s *W* test. Spearman’s Rho (*r*) correlation coefficients were calculated between image quality and acquisition time and work experience and smartphone experience, respectively. The statistical significance level (𝛼) was defined as 0.05 for all of the tests used. The results of the statistical analyses with a *p* value less than 0.05 (*p* < 0.05) were interpreted as statistically significant.

## 3. Results

The smartphone group consisted of twelve consultants, eleven residents, and three medical students. The control providing the reference images should only include trained ophthalmologic photographers. As there was only one photographer meeting our requirements available at our hospital, the control group consisted of one professional ophthalmologic photographer. To eliminate the confounders’ patient compliance and pupil dilation, all of the participants took photos of the same model which limited the number of photos in each group. Detailed demographic data are listed in Table 1.

**Figure 3:**
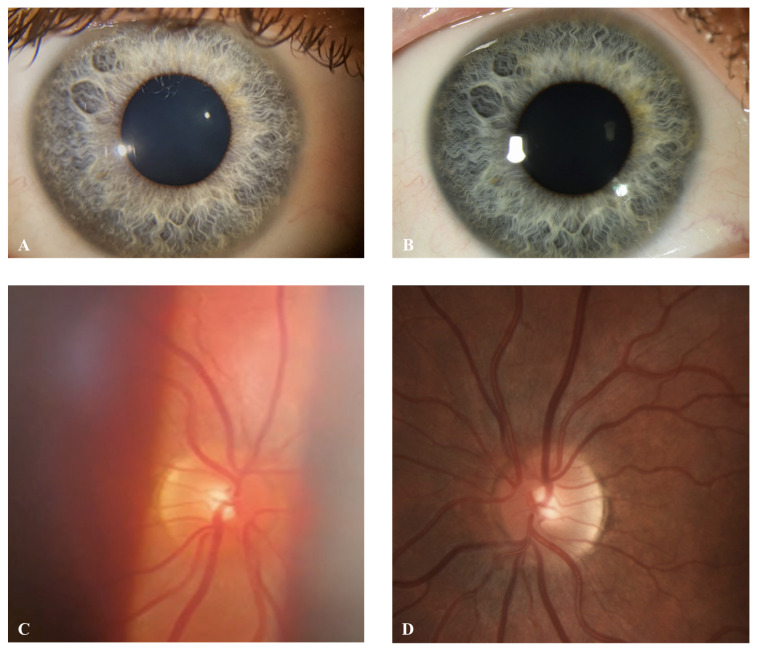
Comparison of smartphone images with photo studio images: (**A**) Anterior segment picture taken by smartphone; (**B**) Anterior segment picture taken by photo studio; (**C**) Posterior segment picture taken by smartphone. **(D**) Posterior segment picture taken by photo studio.

The images by the smartphone group showed an overall quality grading of 7.2/10 of the anterior segment and were hence defined as equally as good as the images by the control group (Figure 3A–D and Table 2). The posterior segment smartphone images did reach a high-quality level but achieved an overall quality score of 6.4, signifying the inferiority to the control group. The Kolmogorow–Smirnow test as well as the Shapiro–Wilk test did not show normal distribution for all variables. Therefore, we decided for tests not requiring normal distribution for further statistical analysis. The calculated Mann–Whitney U test did find a significantly higher quality index for the anterior segment images compared to the posterior segment images within the smartphone group in the overall quality as well as the FOV, clinical interpretability, and artifacts (Table 2).

Apart from the image sharpness of the posterior segment (*p* = 0.015), no significant image quality change between the three takes was noted (all *p* ≥ 0.059) (Table 3). The subjectively rated difficulty to take an image of the anterior and posterior segment as well as the installation of the adapter set decreased significantly within the three takes (Table 3).

The mean acquisition time within the smartphone group did not change significantly between the three attempts (all *p* ≥ 0.558) (Table 3). It was faster for the anterior segment in the smartphone group whereas in the control group, it was faster in taking a posterior segment photo (Table 4). The disturbance of the slit lamp adapter when flipped away during clinical binocular work was low, with a rating of 7.6/10 (10/10 being not disturbing at all) (Table 4). The main feedback we received was that the slit lamp adapter blocked the slit lamp binoculars when trying to adjust for the small pupillary distances (PD) of the examiner. The universal phone adapter when left on the phone while the phone was detached from the slit lamp carried around in the pockets of trousers or the doctor’s coat was rated 5.8/10, implying a slight disturbance (Table 4).

Within the smartphone group, a statistically significant Spearman’s correlation was found between the image quality of posterior segment photos and work experience (Table 5). The other values did not correlate statistically significantly (all *p* ≥ 0.067).

## 4. Discussion

The demographic details of our study cohort show a wide distribution of smartphones as would have been expected in this age group. Every participant had owned a smartphone for at least seven years. Even older smartphones and smartphones of the medium0price segment have a decent built-in camera, making them suitable candidates for slit lamp imaging. Therefore, it is not astonishing that there is are a considerable number of smartphone adapters for slit lamp use already on the market (Table 6) [25,28]. *Roy* et al. evaluated three smartphone adapters: (1) *Magnifi* (Arcturus Labs LLC, Palo Alto, CA, USA), (2) *Skylight* (Skylight Healthcare Systems, Oakland, CA, USA), and (3) *Snapzoom* (HI Resolution Enterprises, Honolulu, HI, USA) in combination with a microscope [29]. They recommend that protective phone cases should be removed before mounting the adapter to the phone to ensure optimal alignment [29]. If mounted and operated correctly, slit lamp image quality depends on hardware such as the smartphone camera sensor’s resolution, the resolution of the optical device (a slit lamp or microscope) as well as the focal length of the smartphone camera system. Current hardware is capable of providing sufficient photo resolutions. The final output image result is dependent on software settings such as autofocus, shutter speed, and manufacturer-specific internal post-processing algorithms when using a compressed image format such as .jpg [29]. Therefore, we restricted our study to one phone type and took the photos with the *MicroREC* camera application as recommended by the adapter manufacturer Custom Surgical. Although the *MicroREC* applications allows manual corrections for white balance (WB), focus, and exposure, we used it in automatic mode to keep the usability as simple and the acquisition time as short as possible. Newer phones with the ability to capture images in raw format will allow and will require more software-based post-processing. *Morales-Leon* et al. showed that stereoscopic image rendering is possible using a software application (*i3DSteroid* by StereoPhoto Maker (Spmaker) by Masuji Suto, Japan, https://stereo.jpn.org (accessed on 20 October 2022)) processing two simultaneously recorded smartphone photos via two parallel smartphone adapters (*Eyecapp* formerly *Cruise Ophthalmic*, Mexico City, Mexico), one on each eyepiece [30]. They evaluated image quality and stereoscopic information based on clinical findings such as trabeculectomy bullae, penetrating keratoplasty (PK), and the optic nerve (ON) [30]. They concluded that stereoscopic images by simultaneous recording of two images with two adapters and later software reconstruction is also possible allowing for better evaluation of clinical findings such as optic disc configuration [30]. Support by artificial intelligence (AI) image analysis algorithms will increase the interpretability of clinical photos [31].

To be applicable not only for research purposes but in clinical daily life, an imaging device must be easy and quick to operate with a steep learning curve. *Roy* et al. found that using a smartphone adapter did slow their workflow as they could not flip away the adapter, thus having to dismount the adapter each time they wanted to use the binoculars [29]. We found smartphone imaging to be quick; for the anterior segment photos, it was even quicker than the control group. Furthermore, we could show with a quick learning curve over the course of only three attempts. We did not evaluate interruptions of the workflow by the adapter during a clinical workday. However, we assume only a small impact as the adapter tested in our study can be flipped aside.

One limitation of our study was the small sample size of the control group which did not allow us to make direct statistical comparisons with the smartphone group. Furthermore, a comparison with previous studies that tested different adapters is hindered as we only included one adapter in our study. However, our market review has revealed that several of the previously evaluated adapters have been discontinued (Table 6). We could have further included and compared do-it-yourself (DIY) adapters made of standard parts such as *Chan* et al. or *Raju* et al.’s suggestions or 3D-printed them based on a computer-aided design (CAD) model as proposed by *Ateya* et al. [32,33,34]. A self-made adapter would face the problem that the product would lack certification (e.g., Conformité Européenne, CE) raising questions of legal liability. The certification process is usually not profitable for low-cost products in small quantities. The impact of software settings and post-processing of different smartphone models should be evaluated in future studies. However, even with highly optimized software image processing, a larger image sensor will always hold the higher potential for image quality as physics cannot be fooled.

## 5. Conclusion

Slit lamp imaging with the presented smartphone adapter provides high quality imaging of the anterior segment. Posterior segment imaging remains challenging in terms of image quality and has a longer learning curve assuming solid fundoscopy skills. The evaluated smartphone adapter constitutes a cost-effective, portable, easy-to-use solution for recording ophthalmic photos and videos. It can facilitate clinical documentation and communication among colleagues and with the patient especially outside normal consultation hours. Direct slit lamp imaging allows for time to be saved and increases the independence of ophthalmologists in patient mobility and in the availability of photographic staff. It is a step further in making medical imaging more widely and readily available.

## Figures and Tables

**Table 1:** Demographic data.

	Smartphone Group	Control Group
Group size (*n*)	Twenty-Six: - Twelve consultants - Eleven residents - Three medical students	1
Age [years] (median (IQR))	32.00 (10.00)	28.00 (0)
Gender ratio female:male	13:13	1:0
Professional experience [years] (median (IQR)) (min–max range)	2.58 (6.80) (0–17)	4.0 ± 0
Smartphone experience [years using a smartphone] (median (IQR)) (min–max range)	100% of participants 13.00 (5.00) (7–21) 52% Apple *iOS* 48% Google *Android*	100% of participants 12.00 (0) (*N/A*) 100% Google *Android*
Personal smartphone competence of (private) photos taken with a smartphone (mean ± SD)	8.00 (2.00)	*N/A*
Number of photos taken with a smartphone [% of participants]	17%: ≤100/year 50%: >100 and ≤1000/year 33%: >1000/year	*N/A*
Number of previous attempts to take a slit lamp photo with a smartphone [% of participants]	46%: 0 attempts 4%: ≥1 and <2 attempts 50%: ≥2 attempts	*N/A*

Legend: IQR, interquartile range defined as Q3−Q1; median, defined as second quartile (Q2, 50th percentile); N/A, not applicable; min, minimum; max, maximum; Q1, first quartile (25th percentile); Q3, third quartile (75th percentile); SD, standard deviation of arithmetic mean.

**Table 2:** Image grading smartphone group.

	Anterior Segment	Posterior Segment	Mann–Whitney U test
Overall quality (median (IQR))	8 (4.00)	7 (5.00)	*p* = 0.019 *
Sharpness (median (IQR))	8 (3.75)	7 (4.50)	*p* = 0.075
Exposure (median (IQR))	7 (4.00)	7 (4.00)	*p* = 0.964
Field of view (median (IQR))	8 (4.00)	6 (5.00)	*p* < 0.001 *
Color (median (IQR))	8 (5.00)	7 (4.50)	*p* = 0.262
Clinical interpretability (median (IQR))	8 (5.00)	7 (5.00)	*p* = 0.007 *
Artifacts	8 (4.75)	6 (4.00)	*p* < 0.001 *

Legend: IQR, interquartile range defined as Q3−Q1; median, defined as second quartile (Q2, 50th percentile); Q1, first quartile (25th percentile); Q3, third quartile (75th percentile); *, statistically significant.

**Table 3:** Comparison of takes in the smartphone group.

	Take 1	Take 2	Take 3	Kendall’s W
**Anterior segment**				
Quality overall (median (IQR))	6.83 (3.06)	7.17 (3.92)	7.33 (3.04)	*p* = 0.060
Quality sharpness (median (IQR))	8.00 (3.00)	7.00 (3.50)	8.00 (3.00)	*p* = 0.450
Quality exposure (median (IQR))	6.50 (3.38)	7.00 (4.50)	7.00 (3.75)	*p* = 0.108
Quality field of view (median (IQR))	7.50 (4.13)	8.50 (2.50)	8.25 (2.38)	*p* = 0.059
Quality color (median (IQR))	7.50 (3.25)	7.50 (2.50)	7.25 (3.38)	*p* = 0.347
Quality clinical interpretability (median (IQR))	7.25 (3.88)	8.00 (3.50)	7.75 (3.88)	*p* = 0.350
Quality artifacts (median (IQR))	7.25 (3.38)	7.50 (3.50)	7.50 (3.75)	*p* = 0.264
Acquisition time [sec] (median (IQR))	143 (67.00)	129 (68.50)	127 (56.75)	*p* = 0.558
Subjective difficulty image acquisition (median (IQR))	8.00 (3.00)	9.00 (2.00)	9.00 (2.00)	*p* < 0.001 *
**Posterior segment**				
Overall quality (median (IQR))	6.00 (5.13)	6.75 (3.00)	7.50 (5.50)	*p* = 0.094
Sharpness (median (IQR))	6.75 (5.13)	7.25 (3.13)	8.25 (4.13)	*p* = 0.015
Exposure (median (IQR))	6.25 (3.88)	7.25 (3.13)	7.00 (4.13)	*p* = 0.788
Field of view (median (IQR))	5.00 (4.38)	5.75 (3.38)	6.75 (3.75)	*p* = 0.284
Color (median (IQR))	6.50 (3.38)	7.00 (2.88)	7.00 (4.13)	*p* = 0.351
Clinical interpretability (median (IQR))	5.75 (5.00)	6.50 (3.00)	7.00 (5.13)	*p* = 0.097
Artifacts (median (IQR))	5.25 (2.88)	6.00 (3.75)	6.25 (4.63)	*p* = 0.149
Acquisition time [sec] (median (IQR))	170 (117.00)	141 (80.00)	143 (78.75)	*p* = 0.622
Subjective difficulty image acquisition (median (IQR))	6.00 (2.50)	7.00 (2.00)	8.00 (2.00)	*p* = 0.002 *
**Subjective difficulty device installation (median (IQR))**	9.00 (1.75)	9.50 (1.00)	10.00 (1.00)	*p* = 0.004 *

Legend: IQR, interquartile range defined as Q3−Q1; median, defined as second quartile (Q2, 50th percentile); Q1, first quartile (25th percentile); Q3, third quartile (75th percentile); *, statistically significant.

**Table 4:** Usability data.

	Smartphone Group	Control Group
Acquisition time [sec] anterior (median (IQR))	133.67 (55.33)	192.00 (20.50)
Acquisition time [sec] posterior (median (IQR))	153.17 (95.83)	140.00 (19.00)
Disturbance of adapter on slit lamp (median (IQR))	8.00 (1.00)	*N/A*
Disturbance of adapter on the phone (median (IQR))	6.00 (4.00)	*N/A*
Complexity of the adapter set (median (IQR))	9.00 (2.00)	*N/A*

Legend: IQR, interquartile range defined as Q3−Q1; median, defined as second quartile (Q2, 50th percentile); Q1, first quartile (25th percentile); Q3, third quartile (75th percentile).

**Table 5:** Correlations in the smartphone group.

	Anterior Segment		Posterior Segment	
Image quality vs. acquisition time	*r* = −0.371	*p* = 0.068	*r* = −0.172	*p* = 0.444
Image quality vs. work experience	*r =* 0.291	*p* = 0.167	*r* = 0.476	*p* = 0.025 *
Image quality vs. smartphone use	*r =* −0.008	*p* = 0.969	*r =* 0.145	*p* = 0.508
Image quality vs. personal smartphone confidence	*r =* 0.097	*p* = 0.644	*r =* −0.087	*p* = 0.694
Acquisition time vs. work experience	*r =* 0.202	*p* = 0.356	*r =* 0.252	*p* = 0.284
Acquisition time vs. smartphone use	*r =* 0.090	*p* = 0.675	*r =* 0.010	*p* = 0.964
Acquisition time vs. personal smartphone confidence	*r =* 0.074	*p* = 0.732	*r =* 0.107	*p* = 0.645

Legend: *p*-value (defined significant when *p* < 0.05); *r*, Spearman’s Rho correlation coefficient; SD, standard deviation of arithmetic mean; *, statistically significant.

**Table 6:** Overview of selected smartphone slit lamp adapters in alphabetical order by brand name, identified by *Google* web searches for the search term *smartphone slit lamp adapter* (as of 9 October 2022) [no financial interest of authors].

Brand	Product	Source (as of October 2022)
Arcturus Labs	Magnifi photoadapter	*Discontinued* (www.arcturuslabs.com (accessed on 20 October 2022))
Celestron	NexYZ universal smartphone adapter	https://www.celestron.de/ce_de/nexyz-universaler-3-achsen-smartphone-adapter.html (accessed on 20 October 2022)
Digital Eye Center	Universal smartphone slit lamp adapter w/ sleeves	https://www.digitaleyecenter.com/product/universal-smartphone-slit lamp-adapter (accessed on 20 October 2022)
Eye2Mobile	PHONEdock	https://www.eye2mobile.com/ (accessed on 20 October 2022)
Eyecapp (*formerly* Cruise Ophthalmic)	Smartphone adapter	*Discontinued* (https://www.eyecapp.com) (accessed on 20 October 2022)
Eye Photo Doc	Smartphone universal case	https://www.eyephotodoc.com/Price_of_Eyephotodoc_iPhone_systems.html (accessed on 20 October 2022)
HI Resolution Enterprises	Snapzoom universal digiscoping adapter	https://www.snapzooms.com/shop/6v3efy0iew39g7e8vbx3tspwjykz95/6v3efy0iew39g7e8vbx3tspwjykz95 (accessed on 20 October 2022)
Keeler	Portable slit lamp iPhone 4 imaging adapter (3010-P-7010)	*Discontinued*
Kowa Optimed	Smartoscope Vario universal smartphone adapter	https://www.kowaoptic.com/de/smartoscope-vario-universal-smartphone-adapter?c=84 (accessed on 20 October 2022)
New Vision	Smartphone photographic adapter	http://www.4vision.cn/P_view.asp?pid=191 (accessed on 20 October 2022)
oDocs	Slit Lamp adapter	https://odocseyecare.shop/products/odocs-slit lamp-surgical-microscope-adapter (accessed on 20 October 2022)
Optimetrics	Smartphone digital slit lamp metal adapter	https://optimetrics.com/home/1265-smart-phone-digital-slit lamp-adapter.html (accessed on 20 October 2022)
Orion Telescopes & Binoculars	SteadyPix telescope photo adapter for iPhones	https://www.telescope.com/Astrophotography/Astrophotography-Accessories/Orion-SteadyPix-Telescope-Photo-Adapter-for-iPhone/c/4/sc/61/p/101445.uts (accessed on 20 October 2022)
Seiler Medical	Smartphone adaptor (IPH-VA)	https://www.seilermicro.com/products/accessories/smart-phone-adaptor (accessed on 20 October 2022)
Skylight Healthcare Systems	SkyLight smartphone adapter	*Discontinued* (https://opticsmag.com/skylight-scope-microscope-cell-phone-adapter (accessed on 20 October 2022))
TigerLens	Smartphone adapter	*Discontinued* (www.tigerlens.com (accessed on 20 October 2022))
TTI Medical	ACCU-BEAM universal smartphone adapter (8100SP)	https://ttimedical.com/products/digital-adapters/smartphone-adapter (accessed on 20 October 2022)
Welch Allyn/Hillrom (now part of Baxter Inc.)	iExaminer adapter for iPhones (11840)	*Discontinued* (https://www.welchallyn.com/en/microsites/iexaminer.html (accessed on 20 October 2022))
Zarf Enterprises	Slit lamp digital camera adapters	http://www.zarfenterprises.com (accessed on 20 October 2022)

Legend: N/A, not applicable.

## Data Availability

The data are not publicly available due to privacy regulations.

## Supplementary Materials

The following are available online at https://www.mdpi.com/article/10.3390/diagnostics13030423/s1, Supplementary Material SM 1: Smartphone Slit Lamp Imaging-Usability and Quality Assessment
