# Supplementary material for: Smartphone Slit Lamp Imaging—Usability and Quality Assessment
Source: Diagnostics (Basel). 2023 Jan 24;13(3):423. doi: 10.3390/diagnostics13030423 (PMC9913954; doi:10.3390/diagnostics13030423)
Supplement: Supplementary file 1 [file diagnostics-13-00423-s001.zip › diagnostics-2015166-supplementary.pdf]

University of Zurich[UZH]

USZ Universitäts Spital Zürich

Forschungsprojekt Augenklinik
PD Dr. med. Sandrine Zweifel | Stv.Klinikdirektorin

Augenklinik

Smartphone Slit Lamp Imaging - Usability and Quality Assessment

Name and surname of the participant:……………………………………
Study ID #:

Demographics

Date of birth:…………………………….. Gender: M / F

Profession / Position in the clinic:

- o Unterassistent / Medical Student
- o Assistenzarzt / Resident
- o Oberarzt / Consultant
- o Photograph of the photo team

- **PREVIOUS USER EXPERIENCE:**

For how many years do you own a private smartphone?	………years<br>(0 = no smartphone)	
Which type of smartphone do you use most privately (brand, operating system [iOS, Android etc.]		
How much do you use your private smartphone for taking private photos?	a) <= 100 photos/year<br>b) >100 to <= 1000 photos/year<br>c) >1000 photos/year	
How satisfied were you with the quality of the private pictures taken with your smartphone?	(0 = not satisfied / 10= Very satisfied)<br>0 1 2 3 4 5 6 7 8 9 10	
Have you already used your smartphone to capture images from a slit lamp?	0 = Never<br>1 = Just once<br>2 = >2 and < 30 times<br>3 = >= 30 times	
How many years (months, days) of experience do you have with slit lamp examinations?	Anterior segment	Fundoscopy
How confident do you feel with slit lamp examinations?<br>(0 = not confident / 10= very confident)<br>0 1 2 3 4 5 6 7 8 9 10	Anterior segment	Fundoscopy
Is it the first time that you see the adapter used in this study?	0 = first time;<br>1 = seen before	
Did you already use a smartphone slit lamp adapter by another company?	0 = no;<br>1 = yes	
If yes, what was the name of the company and the model?	………………………………..	
If no, where did you see this adapter before?	If social media, which one?...................<br><br>Other………………………………………	

Forschungsprojekt Augenklinik
PD Dr. med. Sandrine Zweifel | Stv.Klinikdirektorin

Augenklinik

Usability

Adapter-specific

How would you rate the disturbance due to the presence of the adapter on the slit lamp during a visit:	(0 - Very disturbing / 10 – Not disturbing)<br>0 1 2 3 4 5 6 7 8 9 10
How would you rate the disturbance due to the presence of the adapter on the smartphone during the normal clinical activity:	(0 - Very disturbing / 10 – Not disturbing)<br>0 1 2 3 4 5 6 7 8 9 10
Do you think the adapter could be effective in helping completing your work?	(0 =the device wouldn't help you/<br>10 = the device would be a great help)<br>0 1 2 3 4 5 6 7 8 9 10
How could the device help you in your work?	
Do you think the adapter is unnecessarily complex?	(0 = too complex / 10 = not complex)<br>0 1 2 3 4 5 6 7 8 9 10
Do you think that most people would learn to use this device very quickly?	(0=absolutely not / 10=People will definitely quickly learn)<br>0 1 2 3 4 5 6 7 8 9 10
Do you think your ability to use the device can improve after repeated use of the device?	(0=absolutely not / 10= People will definitely get faster)<br>0 1 2 3 4 5 6 7 8 9 10
What are the main disadvantages of this device?	
What are the aspects of the device that you appreciated the most?	

Forschungsprojekt Augenklinik
PD Dr. med. Sandrine Zweifel | Stv.Klinikdirektorin

Augenklinik

What would you improve in the device?	

FOR GRADERS ONLY:

Quality:

Photo of the pupillary rim (0=not good / 10=very good):

	Smartphone no adapter	Smartphone adapter	Photo studio (photo)
-Sharpness:	0 1 2 3 4 5 6 7 8 9 10	0 1 2 3 4 5 6 7 8 9 10	0 1 2 3 4 5 6 7 8 9 10
-Exposure:	0 1 2 3 4 5 6 7 8 9 10	0 1 2 3 4 5 6 7 8 9 10	0 1 2 3 4 5 6 7 8 9 10
-Field of view:	0 1 2 3 4 5 6 7 8 9 10	0 1 2 3 4 5 6 7 8 9 10	0 1 2 3 4 5 6 7 8 9 10
-Reliability of the colors:	0 1 2 3 4 5 6 7 8 9 10	0 1 2 3 4 5 6 7 8 9 10	0 1 2 3 4 5 6 7 8 9 10
-Interpretability of the Pictures:	0 1 2 3 4 5 6 7 8 9 10	0 1 2 3 4 5 6 7 8 9 10	0 1 2 3 4 5 6 7 8 9 10
-Presence of artifacts	0 1 2 3 4 5 6 7 8 9 10	0 1 2 3 4 5 6 7 8 9 10	0 1 2 3 4 5 6 7 8 9 10
Overall quality satisfaction:	0 1 2 3 4 5 6 7 8 9 10	0 1 2 3 4 5 6 7 8 9 10	0 1 2 3 4 5 6 7 8 9 10
Image resolution:			

Photo of the optic disc (0=not good / 10=very good):

	Smartphone no adapter	Smartphone adapter	Photo studio (photo)
-Sharpness:	0 1 2 3 4 5 6 7 8 9 10	0 1 2 3 4 5 6 7 8 9 10	0 1 2 3 4 5 6 7 8 9 10
-Exposure:	0 1 2 3 4 5 6 7 8 9 10	0 1 2 3 4 5 6 7 8 9 10	0 1 2 3 4 5 6 7 8 9 10
-Field of view:	0 1 2 3 4 5 6 7 8 9 10	0 1 2 3 4 5 6 7 8 9 10	0 1 2 3 4 5 6 7 8 9 10
-Reliability of the colors:	0 1 2 3 4 5 6 7 8 9 10	0 1 2 3 4 5 6 7 8 9 10	0 1 2 3 4 5 6 7 8 9 10
-Interpretability of the Pictures:	0 1 2 3 4 5 6 7 8 9 10	0 1 2 3 4 5 6 7 8 9 10	0 1 2 3 4 5 6 7 8 9 10
-Presence of artifacts	0 1 2 3 4 5 6 7 8 9 10	0 1 2 3 4 5 6 7 8 9 10	0 1 2 3 4 5 6 7 8 9 10
Overall quality satisfaction:	0 1 2 3 4 5 6 7 8 9 10	0 1 2 3 4 5 6 7 8 9 10	0 1 2 3 4 5 6 7 8 9 10
Image resolution:			
